# Relationship between area mortgage foreclosures, homeownership, and cardiovascular disease risk factors: The Hispanic Community Health Study/Study of Latinos

**DOI:** 10.1186/s12889-019-6412-2

**Published:** 2019-01-17

**Authors:** Earle C. Chambers, David B. Hanna, Simin Hua, Dustin T. Duncan, Marlene Camacho-Rivera, Shannon N. Zenk, Jessica L. McCurley, Krista Perreira, Marc D. Gellman, Linda C. Gallo

**Affiliations:** 1 Department of Family & Social Medicine, Albert Einstein College of Medicine, 1300 Morris Park Avenue, Harold and Muriel Block Building, Rm. 409, Bronx, NY 10461 USA; 2 Department of Epidemiology & Population Health, Albert Einstein College of Medicine, Bronx, NY 10461 USA; 3 NYU Spatial Epidemiology Laboratory, Department of Population Health, New York University School of Medicine, New York, NY 10016 USA; 4 Department of Community Health and Social Medicine, City University of New York School of Medicine, New York, NY 10031 USA; 5 Department of Health Systems Science, University of Illinois at Chicago College of Nursing, Chicago, IL 60610 USA; 6 Department of Medicine, Massachusetts General Hospital/Harvard Medical School, Boston, MA 02114 USA; 7 Department of Social Medicine, University of North Carolina, Chapel Hill, NC 27599-7240 USA; 8 Behavioral Medicine Research Center, Department of Psychology, University of Miami, Miami, FL 33136 USA; 9 Department of Psychology, San Diego State University, San Diego, CA 92120 USA

**Keywords:** Cardiovascular disease, Housing, Foreclosure, Homeownership

## Abstract

**Background:**

The risk of mortgage foreclosure disproportionately burdens Hispanic/Latino populations perpetuating racial disparities in health. In this study, we examined the relationship between area-level mortgage foreclosure risk, homeownership, and the prevalence of cardiovascular disease risk factors among participants of the Hispanic Community Health Study/Study of Latinos (HCHS/SOL).

**Methods:**

HCHS/SOL participants were age 18–74 years when recruited from four U.S. metropolitan areas. Mortgage foreclosure risk was obtained from the U.S. Department of Housing and Urban Development. Homeownership, sociodemographic factors, and cardiovascular disease risk factors were measured at baseline interview between 2008 and 2011. There were 13,856 individuals contributing to the analysis (median age 39 years old, 53% female).

**Results:**

Renters in high foreclosure risk areas had a higher prevalence of hypertension and hypercholesterolemia but no association with smoking status compared to renters in low foreclosure risk areas. Renters were more likely to smoke cigarettes than homeowners.

**Conclusion:**

Among US Hispanic/Latinos in urban cities, area foreclosure and homeownership have implications for risk of cardiovascular disease.

## Background

Racial residential segregation made Hispanic/Latino and black households particularly vulnerable to predatory lending practices and these populations were thus hardest hit by the U.S. housing crisis beginning in 2007 [1]. Whether the housing crisis placed an additional burden on cardiovascular health in the Hispanic/Latino population is unclear. Hypertension, high cholesterol, and smoking are referred to by the Centers for Disease Control and Prevention (CDC) as key risk factors for heart disease [2]. Nearly half of U.S. adults have at least one of these heart disease risk factors [3]. Prior studies examining the relationship between foreclosure and cardiovascular disease risk have included body mass index (BMI) [4–6], blood pressure [7–9], fasting glucose [7], mental health [10], and risk for hospitalization for heart attack and stroke [9, 11, 12]. These results, however, have been inconsistent. For example, Arcaya and colleagues [8] showed that adults living in close proximity to a foreclosed property were more likely to have elevated blood pressure, whereas Christine et al. [7] showed a small inverse relationship between foreclosures in a neighborhood and blood pressure. Studies of associations of area foreclosures and BMI are similarly inconsistent [4–6]. Inconsistent findings among these studies may reflect differences in measurement of foreclosures at the neighborhood levels or possibly differing mechanisms driving the association between foreclosures and various cardiovascular disease risk factors.

As research further uncovers the mechanisms linking foreclosures to health, examining the role of homeownership can be an important step in guiding policy. Homeowners are generally considered the most vulnerable during a foreclosure crisis but studies showing an association between neighborhood foreclosures and health suggest that both homeowners and renters can be affected by high foreclosures in the area. [4, 11, 12]. A high level of neighborhood foreclosures may influence health outcomes by reflecting conditions that contribute to environmental-related stress of residents [8, 10]; limited access to health care [11, 12]; and/or limited access to resources for a healthy diet and physical activity for residents [13]. More research is needed to confirm prior findings of the association of neighborhood foreclosures and cardiovascular disease. These studies should characterize the risk among homeowners and renters and examine a wider range of cardiovascular disease risk factors such as high cholesterol and cigarette smoking.

Despite a growing literature on the associations between neighborhood foreclosure and health [14], as well as homeownership and health [15], existing studies examining cardiovascular disease risk factors suffer from a variety of limitations. First, most studies do not examine more nuanced associations between neighborhood foreclosure, homeownership, and health. For example, among residents living in proximity to foreclosed properties, it is not clear whether residents who rent are as likely to show the poor health-related association of living in a high foreclosure risk neighborhood as residents who own their own home. Second, few studies include health behaviors associated with cardiovascular disease such as smoking. Third, many studies have not included large samples of racial and ethnic minorities, including Hispanic/Latinos – the largest ethnic minority population in the U.S. Fourth, many studies focus on single cities or limited geographical areas, which limits generalizability. The purpose of this study was to examine the relationship between neighborhood foreclosure risk, homeownership, and cardiovascular disease risk factors - i.e., hypertension, hypercholesterolemia, cigarette smoking - among Hispanic/Latino adults living in 4 major metropolitan areas in the U.S. We further examined whether the association of neighborhood foreclosure risk on cardiovascular disease risk factors differed between homeowners and renters, rarely addressed in previous research.

## Methods

### Study population and design

The Hispanic Community Health Study/Study of Latinos (HCHS/SOL) is a community-based prospective cohort study of 16,415 self-identified Hispanic/Latino persons aged 18–74 years at screening from randomly selected households in four U.S. field centers (Chicago, IL; Miami, FL; Bronx, NY; San Diego, CA) with baseline examination (2008 to 2011) and yearly telephone follow-up assessment. The goals of the HCHS/SOL, sample design, and cohort selection have been previously described [16, 17]. The baseline clinical examination included comprehensive biological (e.g., anthropometrics, blood draw), behavioral (e.g., tobacco use assessed by self-report), and socio-demographic (e.g., socioeconomic status, nativity) assessments. The Institutional Review Board at each field center approved the study. All participants gave written informed consent in either English or Spanish.

### Exposures of interest

#### Mortgage foreclosure risk

In 2008, the U.S. Department of Housing and Urban Development (HUD) created a novel mortgage foreclosure risk metric which estimates mortgage foreclosure risk for the year 2007 and the first six months of 2008 as a function of area decline in home values as of June 2008; unemployment rate as of June 2008; and high cost mortgage loans between 2004 and 2006. The mortgage foreclosure risk metric is estimated at the census tract level and reflects areas in the country that have started or could potentially become areas of abandonment and disinvestment. This measure was used to inform where state and local resources should be targeted to stabilize neighborhoods and stem the decline of house values of homes in these areas. More details on the methodology HUD used to calculate mortgage foreclosure risk is available on the HUD website [18].

#### Homeownership

Homeownership was determined by a question asked during the baseline HCHS/SOL visit: Is your house, apartment, or mobile home… (1) “Owned by you or someone in the household free and clear --- without a mortgage or loan”; (2) “Owned by you or someone in the household--- with a mortgage or loan”; (3) “Rented”; or (4) occupied without rent. In order to be consistent with other studies that do not distinguish between mortgage status among owners, both of the ‘owned’ categories were combined into one category and compared with renters. [19, 20]

### Cardiovascular disease risk factors

Each cardiovascular disease risk factor was measured during the baseline clinic visit of HCHS/SOL participants. Three seated blood pressure measurements were obtained after a 5-min rest period using an automatic sphygmanometer. The average of the second and third measurement was used in analysis. Hypertension was defined as a systolic blood pressure ≥ 140 mmHg, diastolic blood pressure ≥ 90 mmHg, and/or receiving antihypertensive medication. Hypercholesterolemia was defined as total cholesterol ≥240 mg/gL, LDL cholesterol ≥160 mg/ dL, or HDL cholesterol < 40 mg/ dL or receiving cholesterol lowering medications. Cigarette smoking was categorized as never, former, and current use.

### Covariates

Participants’ height was measured to the nearest centimeter and body weight to the nearest 0.1 kg. BMI was calculated as weight in kilograms divided by height in meters squared. BMI categories were defined as underweight (< 18.5 kg/m^2^), normal weight (18.5–24.9 kg/m^2^), overweight (25.0–29.9 kg/m^2^), and obese (≥ 30.0 kg/m^2^). *Potential confounders*. Socio-demographic characteristics self-reported during the baseline exam included: age, sex, household income, education, employment, nativity (foreign-born vs. US-born), and Hispanic/Latino background. Neighborhood percent poverty was defined as the percentage of families per census tract (CT) whose income in the past 12 months was below the poverty line, based on data from 2007 to 2011 American Community Survey 5-year estimates [21, 22].

### Statistical analyses

All participants of the HCHS/SOL cohort with complete information for study variables were included in the current analysis (*n* = 13,856) with several specific exceptions. Residents indicating that they occupy their home without paying rent were excluded from analysis (*n* = 422). Since we were interested in examining risk factor for cardiovascular disease and to reduce the likelihood of reverse causation of residents with pre-existing cardiovascular disease preferentially residing in high foreclosure areas, participants with preexisting cardiovascular disease at the baseline interview were also excluded from analysis (*n* = 1166). Preexisting cardiovascular disease included prevalent coronary heart disease, defined as self-report of history of heart attack or procedure (angioplasty, stent, bypass) or electrocardiogram showing old myocardial infarction; or cerebrovascular disease, defined as self-reported medical history of stroke, mini-stroke or transient ischemic attack, or carotid revascularization or balloon angioplasty or surgery to the arteries in the neck at baseline assessment.

A mortgage foreclosure risk value was attributed to each participant based on his or her residential census tract. The mortgage foreclosure risk variable was linked based on 2000 census tract boundaries, whereas the percent poverty variable was linked to each participant’s census tract based on 2010 census tract boundaries. In this study, 97% of addresses were successfully geocoded. Participants not able to be geocoded were dropped from the analysis (*n* = 551).

We computed descriptive statistics (e.g. proportions) across all study variables. We initially compared all study variables including homeownership across tertiles of mortgage foreclosure risk. Poisson regression models were used to estimate prevalence ratios (PR) with 95% confidence intervals (95% CI) for hypertension, hypercholesterolemia, and smoking by homeownership status and mortgage foreclosure risk, with robust variance estimation used to account for clustering by census tract. Additional stratified analyses were done to examine the association of mortgage foreclosure risk with cardiovascular disease risk factors by homeownership status. Analyses were primarily adjusted for age, sex, education level, employment status, income level, nativity, Hispanic/Latino group, and percent poverty level.

All reported values (means, prevalences, and prevalence ratios) were weighted to account for the disproportionate selection of the sample and to partially adjust for any bias due to differential nonresponse in the selected sample at the household and individual levels. The adjusted weights were also trimmed to limit precision losses due to the variability of the adjusted weights, and calibrated to the 2010 Census characteristics by age, sex, and Hispanic/Latino background in each field site’s target population. All analyses also account for cluster sampling and the use of stratification in sample selection.

Statistical significance was determined at the *P* < 0.05 level. All analyses were performed using SAS 9.4 software (SAS Institute, Cary, NC) and SUDAAN software Release 11.0 (RTI International, Research Triangle Park, NC).

## Results

There were 13,856 HCHS/SOL participants whose data contributed to the study. Table 1 shows the distribution of baseline characteristics of HCHS/SOL participants by tertile of neighborhood mortgage foreclosure risk. Most HCHS/SOL participants lived in a rental unit (74%). A higher percentage of renters lived in high mortgage foreclosure risk areas, than low mortgage foreclosure risk areas; and more homeowners lived in low mortgage foreclosure risk areas than in high mortgage foreclosure risk areas. Participants who identified as Cuban were largely located in areas of high foreclosure risk, while participants who identified as Mexican were largely in areas of low and medium foreclosure risk (Table 1). The census tract-level correlation between mortgage foreclosure risk and percent poverty was *r* = 0.16 (*P* < 0.01), indicating that high mortgage foreclosure risk areas were not uniformly congruent with high poverty areas.

**Table 1:** Demographic, homeownership status, and health characteristics of the HCHS/ SOL cohort by census tract-level mortgage foreclosure risk

	Census tract-level Mortgage Foreclosure Risk							
	Overall		Low		Medium		High	
	N	Median (IQR) or % (95% CI)	N	Median (IQR) or % (95% CI)	N	Median (IQR) or % (95% CI)	N	Median (IQR) or % (95% CI)
Foreclosure rate	13, 851	7.5 (6.2, 9.5)	4444	5.8 (5.0, 6.3)	4737	7.5 (7.0, 8.0)	4670	10.5 (9.2, 11.2)
Homeownership								
Renters	9935	74.1 (71.8, 76.4)	3023	69.8 (64.3, 74.8)	3260	73.3 (69.8, 76.5)	3652	79.0 (75.9, 81.9)
Owners	3916	25.8 (23.6, 28.2)	1421	30.2 (25.2, 35.7)	1477	26.7 (23.5, 30.3)	1018	21.0 (18.1, 24.1)
Demographics								
Age Categories, years								
18–44	5919	61.8 (60.3, 63.3)	1887	62.5 (59.8, 65.1)	2080	65.6 (63.1, 68.0)	1952	57.9 (55.4, 60.4)
45–64	6970	30.8 (29.6, 32.0)	2213	31.0 (28.9, 33.2)	2348	28.9 (26.7, 31.1)	2409	32.3 (30.4, 34.3)
65+	962	7.4 (6.7, 8.2)	344	6.6 (5.4, 8.0)	309	5.5 (4.6, 6.6)	309	9.8 (8.5, 11.4)
Sex								
Female	8379	52.6 (51.5, 53.8)	2725	53.9 (51.8, 56.0)	2895	51.7 (49.5, 54.0)	2759	52.1 (50.4, 53.9)
Male	5472	47.4 (46.2, 48.6)	1719	46.1 (44.0, 48.3)	1842	48.3 (46.0, 50.5)	1911	47.9 (46.1, 49.6)
Education								
< High school	5133	31.4 (29.9, 32.9)	1709	31.5 (28.6, 34.6)	1872	34.3 (31.9, 36.7)	1552	28.8 (26.6, 31.2)
High school	3622	28.5 (27.3, 29.7)	1097	26.2 (24.2, 28.4)	1229	29.3 (27.0, 31.6)	1296	29.9 (28.1, 31.8)
> High school	5096	40.2 (38.5, 41.9)	1638	42.3 (38.7, 46.0)	1636	36.5 (34.1, 38.9)	1822	41.3 (38.7, 43.9)
Annual Income, $								
< 20,000	6001	41.2 (39.4, 42.9)	1793	37.4 (34.0, 40.9)	1952	40.9 (38.0, 43.8)	2256	44.9 (42.5, 47.4)
20,000-50,000	5404	38.3 (36.9, 39.8)	1687	37.6 (35.3, 39.9)	2029	41.9 (39.4, 44.4)	1688	36.1 (33.6, 38.6)
> 50,000	1390	12.1 (10.6, 13.8)	658	18.1 (14.8, 22.0)	466	11.1 (9.4, 13.1)	266	7.2 (6.0, 8.7)
Don’t Know/Refused	1056	8.4 (7.6, 9.3)	306	6.9 (5.9, 8.2)	290	6.1 (5.1, 7.3)	460	11.8 (10.2, 13.6)
Hispanic/Latino background								
Dominican	1202	9.4 (8.1, 10.9)	668	15.5 (12.6, 18.9)	363	8.9 (6.9, 11.5)	171	4.1 (2.4, 6.7)
Central American	1526	7.8 (6.7, 9.0)	316	4.8 (3.9, 5.9)	304	5.1 (3.9, 6.7)	906	12.8 (10.4, 15.7)
Cuban	2036	20.6 (17.4, 24.2)	67	2.2 (1.5, 3.1)	170	6.3 (4.4, 8.9)	1799	50.0 (43.7, 56.3)
Mexican	5606	38.3 (35.1,41.7)	1788	44.4 (38.9, 50.1)	2823	55.7 (50.5, 60.8)	995	18.0 (13.1, 24.3)
Puerto Rican	2128	14.9 (13.4, 16.5)	1074	22.6 (19.5, 26.0)	708	16.0 (13.5, 18.8)	346	6.7 (4.9, 8.9)
South American	933	5.0 (4.4, 5.7)	353	5.6 (4.5, 6.9)	249	4.1 (3.3, 5.2)	331	5.2 (4.2, 6.5)
Mixed/other	420	4.0 (3.5, 4.6)	178	4.9 (4.0, 6.0)	120	3.9 (2.9, 5.3)	122	3.2 (2.5, 4.1)
Employment								
Retired	1128	7.0 (6.3, 7.8)	443	7.3 (6.0, 8.8)	360	5.8 (4.9, 6.9)	325	7.7 (6.6, 9.1)
Unemployed	5429	40.8 (39.4, 42.2)	1603	38.4 (36.1, 40.9)	1818	39.9 (37.5, 42.4)	2008	43.8 (41.4, 46.1)
Part-time	2435	17.6 (16.7, 18.5)	794	18.1 (16.6, 19.8)	884	19.2 (17.7, 20.8)	757	15.7 (14.2, 17.4)
Full-time	4859	34.6 (33.3, 36.0)	1604	36.1 (33.8, 38.6)	1675	35.1 (32.7, 37.6)	1580	32.8 (30.8, 34.9)
Nativity								
Foreign Born	11,432	77.2 (75.5, 78.8)	3411	70.0 (67.6, 72.3)	3799	73.9 (71.2, 76.5)	4222	86.7 (83.7, 89.2)
US Born (50 States and District of Columbia)	2419	22.8 (21.2, 24.6)	1033	30.0 (27.7, 32.4)	938	26.1 (23.5, 28.8)	448	13.3 (10.8, 16.3)
Tertiles of % poverty								
Low	4742	34.3 (30.4, 38.5)	1934	46.6 (39.3, 54.0)	2041	38.8 (31.5, 46.5)	767	19.1 (14.8, 24.3)
Medium	4603	30.8 (26.8, 35.2)	1248	24.1 (18.6, 30.6)	1518	32.1 (25.2, 39.8)	1837	36.2 (28.6, 44.6)
High	4506	34.8 (30.8, 39.1)	1262	29.4 (23.7, 35.7)	1178	29.2 (23.0, 36.2)	2066	44.7 (36.5, 53.2)
Health								
Hypertension	3806	21.8 (20.7, 23.1)	1243	19.4 (17.6, 21.3)	1173	18.8 (16.9, 20.8)	1390	26.7 (24.7, 28.9)
Hypercholesterolemia	6064	40.6 (39.3, 41.8)	1903	38.1 (35.9, 40.3)	2015	39.1 (36.9, 41.3)	2146	44.2 (42.2, 46.2)
Current Cigarette Use	2616	20.7 (19.6, 21.8)	753	18.4 (16.7, 20.1)	833	19.9 (18.1, 21.9)	1030	23.5 (21.7, 25.5)
BMI, kg/m^2^								
Underweight (< 18.5)	106	1.1 (0.9, 1.5)	34	1.5 (0.9, 2.3)	27	0.6 (0.4, 1.0)	45	1.2 (0.8, 1.8)
Normal (18.5–24.9)	2750	22.7 (21.7, 23.8)	878	22.7 (21.0, 24.5)	911	21.8 (19.9, 23.8)	961	23.5 (21.9, 25.1)
Overweight (25–29.9)	5244	37.6 (36.3, 38.8)	1681	38.0 (35.6, 40.4)	1810	37.9 (35.6, 40.3)	1753	36.9 (35.238.6)
Obese (≥30)	5751	38.6 (37.3, 39.9)	1851	37.9 (35.5, 40.3)	1989	39.6 (37.3, 42.1)	1911	38.4 (36.5, 40.4)
Center								
Bronx	3204	26.8 (24.1, 29.7)	1900	46.1 (39.7, 52.7)	987	27.5 (21.8, 34.0)	317	8.0 (4.9, 12.8)
Chicago	3662	16.7 (14.7, 18.8)	1472	21.0 (16.5, 26.3)	1301	19.9 (15.0, 26.0)	889	9.8 (6.3, 15.0)
Miami	3543	30.3 (26.2, 34.8)	94	2.9 (1.7, 4.7)	347	10.9 (7.8, 15.0)	3102	72.4 (64.8, 79.0)
San Diego	3442	26.3 (23.0, 29.8)	978	30.0 (23.1, 38.0)	2102	41.7 (35.1, 48.6)	362	9.8 (5.7, 16.2)

Census tract-level Mortgage Foreclosure Risk: Low: foreclosure rates ≤6.65%; Medium: 6.65–8.44%; High: > 8.44% in the HCHS/SOL cohort

Hypertension is defined as: systolic or diastolic BP is greater than or equal to 140/90 mmHg or if the participant self-reported as currently taking antihypertensive mediations. Participants without a blood pressure measurement and no medication use were assumed to be not hypertensive

Dyslipidemia is defined as: total cholesterol ≥240 or Low Density Lipoprotein cholesterol ≥160 or High Density Lipoprotein cholesterol < 40 or use of lipid lowering drugs

Crude PR showed that each increase in tertile of foreclosure was associated with an increasing trend in prevalence of hypertension and current smoking status and a decreasing trend in hypercholesterolemia [Table 2]. In adjusted models, high foreclosure risk was positively associated with prevalence of hypertension and hypercholesterolemia among renters but not among homeowners [Table 2]. Homeownership was associated with a lower prevalence of smoking (APR: 0.83; 95% CI: 0.72–0.96) but was not associated with hypertension (APR: 1.08; 95% CI: 0.98–1.18), or hypercholesterolemia (APR: 0.98; 95% CI: 0.90–1.06) in adjusted models including foreclosure risk, BMI, and confounding variables.

**Table 2:** Multivariable Poisson regression analysis of census tract-level mortgage foreclosure risk and prevalence of cardiovascular disease risk factors stratified by homeownership status among HCHS/SOL participants

	Owners		Renters	
	Crude PR (95% CI)	Adjusted^a^ PR (95% CI)	Crude PR (95% CI)	Adjusted^a^ PR (95% CI)
Census tract-level mortgage foreclosure risk				
Hypertension				
High	1.09 (0.87, 1.37)	0.87 (0.68, 1.11)	1.52 (1.31, 1.76)	1.25 (1.08, 1.46)
Medium	0.90 (0.71, 1.14)	0.99 (0.84, 1.17)	1.01 (0.86, 1.20)	1.10 (0.96, 1.28)
Low (referent)	–	–	–	–
Hypercholesterolemia				
High	0.89 (0.75, 1.05)	0.94 (0.80, 1.11)	1.04 (0.95, 1.14)	1.12 (1.01, 1.24)
Medium	0.98 (0.85, 1.14)	0.98 (0.84, 1.13)	1.14 (1.03, 1.25)	1.01 (0.92, 1.11)
Low (referent)	–	–	–	–
Current Smoking				
High	1.27 (0.97, 1.67)	1.16 (0.80, 1.68)	1.10 (0.95, 1.27)	1.12 (0.95, 1.31)
Medium	1.22 (0.91, 1.63)	0.95 (0.72, 1.25)	1.02 (0.86, 1.20)	1.06 (0.91, 1.24)
Low (referent)	–	–	–	–

*CI* confidence interval, *PR* prevalence ratio. Census tract-level mortgage foreclosure risk: Low: foreclosure rates ≤6.65%; Medium: 6.65–8.44%; High: > 8.44% in the HCHS/SOL cohort. ^a^ Adjusted for age, sex, education, income, ethnicity, body mass index, cigarette use (except in smoking model), nativity, employment, and neighborhood poverty

## Discussion

The HUD mortgage foreclosure risk metric identifies at-risk neighborhoods on the verge of economic instability and crisis. It is a measure that captures areas starting to decline providing a window to prevention where policy may influence cardiovascular disease. Using a sample of Hispanics/Latinos sheds light on the health consequences of a largely growing demographic in the U.S. hardest hit by the 2007 housing crisis. Our results showing a significant association of area foreclosure risk with hypertension and hypercholesterolemia among renters but not homeowners suggest that renters are particularly vulnerable in areas where the risk for foreclosures is high. The null finding among homeowners may suggest that residents owning their homes are better able to weather the economic downturn than renters. This is consistent with data showing that homeowners have stronger social ties within the communities that they live in and are better able to access the resources in their neighborhood that promote healthier lifestyle choices and overall wellbeing [23–25]. In our study, we also showed that renters were more likely to be current cigarette smokers than homeowners. This is consistent with reports showing smoking risk to be almost three times more likely among renters than homeowners [26]. Renting, in this study, may indicate a less stable housing environment; and living in less stable housing undermines prioritizing healthy behaviors [27, 28].

Saegert proposes a model where home foreclosures also contribute to racial disparities in housing and place racial and ethnic minorities in housing niches where once a household is in this niche they are exposed to cumulative hazards that affect current and future generations [29, 30]. This is an interesting approach to understanding the health consequences of area foreclosures as a contextual exposure influencing access to resources over time. More research is needed to further elucidate the pathways that might link neighborhood foreclosures and cardiometabolic disease. Considering the intersection of foreclosure, homeownership, and health can inform housing policies with the potential to broaden public health impact and reduce racial and ethnic and socio-economic status-related health disparities [31, 32].

The strength of our foreclosure metric is that it is consistent with federal guidelines used to determine the allocation of resources to communities in most need. The health consequences of the foreclosure crisis that preceded the Great Recession affected renters as well as homeowners. [9] In our study, we posit that neighborhood foreclosure risk is a useful measure of the neighborhood cost of economic decline particularly as it relates to housing insecurity, which can affect all of those living in an area, renters and homeowners alike. Foreclosure risk as measured in this study has some precedent in its use in understanding adverse health outcomes. In a sample of breast cancer survivors, Schootman et al. [20] used a HUD predicted measure of area foreclosures to show that women living in high foreclosure risk areas are more likely to report fair-poor health than women in low foreclosure risk areas. This suggests that residents can begin to feel the distress of living in an at-risk area prior to an actual increase in foreclosure rates. This measure is directly applicable to federal housing-related policies that use this metric to distribute aid to vulnerable communities.

### Limitations

This study is cross-sectional; therefore, it is not possible to determine the temporal relationship between foreclosure, homeownership, and cardiovascular disease risk factors. By limiting our analysis to participants without cardiovascular disease, however, we sought to limit reverse causation where participants with illness were more likely to be in a neighborhood with more foreclosure risk and less likely to be a homeowner. Furthermore, all analyses assume no residential movement between mortgage foreclosure risk assessment and the baseline interview/clinical assessment of HCHS/SOL participants. It may be that residents who moved had foreclosure exposure inconsistent with the census tract associated with their health-related outcomes at the time of clinical measurement. While we are unable to determine how many participants this affects, approximately 14% of Hispanics/Latinos in the U.S. change residence in a given year, compared to 11% of non-Hispanic whites [33]. Healthier people with more resources to buffer the negative influence of adverse exposures in their neighborhood may selectively move to better neighborhoods with lower risk of foreclosure. As a result, the data showing better health outcomes in neighborhoods with a lower foreclosure risk could suffer from some selection bias. This is a common limitation of cross-sectional area-level studies that can best be addressed with a longitudinal study design. Another limitation of our study is that our data do not allow us to determine which residents are personally undergoing a foreclosure at the time of data collection. Prior research indicates that these residents may be more likely to experience stress due to their impending foreclosure status [34]. It is possible that the stress of an impending eviction may limit an individual’s ability to access health promoting resources.. Our data only allow an examination of the relationship between living in a place with high foreclosure risk and CVD risk factors and is unable to determine the association of a personal foreclosure experienced by residents with CVD risk. Finally, as this study was conducted among Hispanic/Latino adults in four urban metropolitan areas, these results might not be generalizable to other populations. Additional research is needed to examine differences in foreclosure experiences and health affects among diverse racial and ethnic groups.

## Conclusion

There are substantial burdens of cardiovascular disease risk factors in the U.S. among all major Hispanic/Latino background groups [35], and greater risks in low-income individuals [3]. As the pathways that contribute to these racial/ethnic and SES disparities in cardiovascular health are uncovered, housing has been identified as an important upstream social determinant that requires interdisciplinary interventions to address [36]. The growing evidence including the results from this study show that home mortgage foreclosures can negatively affect community health. Public health practitioners are increasingly looking outside of health care to housing policy to address many social and economic determinants of health [36, 37]. Housing policies that provide pathways to stable housing for homeowners and renters may bolster cardiovascular disease prevention campaigns within neighborhoods. For example, Making Home Affordable (MHA) programs are resources available to homeowners to help keep them in their homes should they be faced with the possibility of a foreclosure. Among renters, preventing eviction and displacement by implementing legislation that provides rental relief for tenants that pay more than 30% of their gross income on rent and utilities such as that proposed under the Rental Relief Act; or providing legal representation in housing court for low-income residents as is being done in New York City can be useful strategies to stabilize housing for vulnerable residents. Providing opportunities for residents to stay securely housed has the potential to curb the adverse health outcomes that accompany unstable housing.

## Abbreviations

95% CI: 95% Confidence Interval
APR: Adjusted Prevalence Ratio
BMI: Body Mass Index
CT: Census Tract
HCHS/SOL: Hispanic Community Health Study/Study of Latinos
HUD: U.S. Department of Housing and Urban Development
PR: Prevalence Ratios
